# Differences in Liver TFAM Binding to mtDNA and mtDNA Damage between Aged and Extremely Aged Rats

**DOI:** 10.3390/ijms20102601

**Published:** 2019-05-27

**Authors:** Guglielmina Chimienti, Anna Picca, Flavio Fracasso, Emanuele Marzetti, Riccardo Calvani, Christiaan Leeuwenburgh, Francesco Russo, Angela Maria Serena Lezza, Vito Pesce

**Affiliations:** 1 Department of Biosciences, Biotechnologies and Biopharmaceutics, University of Bari Aldo Moro, Via Orabona 4, 70125 Bari, Italy; guglielminaalessandra.chimienti@uniba.it (G.C.); flavio.fracasso@uniba.it (F.F.); angelamariaserena.lezza@uniba.it (A.M.S.L.); 2 Università Cattolica del Sacro Cuore, L.go A. Gemelli 1, 00168 Rome, Italy; anna.picca1@gmail.com (A.P.); riccardo.calvani@gmail.com (R.C.); 3 Fondazione Policlinico Universitario “Agostino Gemelli” IRCCS; L.go F. Vito 8, 00168 Rome, Italy; emanuele.marzetti@policlinicogemelli.it; 4 Department of Aging and Geriatric Research, Institute on Aging, Division of Biology of Aging, University of Florida, Gainesville, FL 32611, USA; cleeuwen@ufl.edu; 5 Laboratory of Nutritional Pathophysiology, National Institute of Gastroenterology “S. de Bellis”, Research Hospital, Castellana Grotte 70013, Italy; francesco.russo@irccsdebellis.it

**Keywords:** rat liver, longevity, mtDNA content, mtDNA common deletion, TFAM binding, 8-oxodG incidence

## Abstract

While mitochondrial dysfunction is acknowledged as a major feature of aging, much less is known about the role of mitochondria in extended longevity. Livers from aged (28-month-old) and extremely aged (32-month-old) rats were analyzed for citrate synthase activity, mitochondrial transcription factor A (TFAM) amount, mitochondrial DNA (mtDNA), and 4.8 Kb “common deletion” contents. None of the assayed parameters differed significantly between age groups. TFAM-binding to mtDNA and the incidence of 8-oxo-deoxyguanosine in specific mtDNA regions, encompassing the origins of mtDNA replication (D-loop and Ori-L) and the 16-bp long direct repeat 1 (DR1) of the 4.8 Kb deletion, were determined. A decrease in TFAM binding was unveiled at all regions in extremely aged in comparison with aged rats. Reduced incidence of oxidized purines at all assayed regions was detected in 32-month-old rats compared with the 28-month-old group. A significant positive correlation between the incidence of 8-oxo-deoxoguanosine and TFAM-bound mtDNA was found at D-Loop and Ori-L regions only in 28-month-old rats. The absence of such correlation in 32-month-old rats indicates a different, fine-tuned regulation of TFAM binding in the two age groups and supports the existence of two different paces in aging and extended aging.

## 1. Introduction

Mitochondrial dysfunction is a well-known feature of the aging process [1,2,3,4] that impinges on both organelle’s maintenance (mitochondrial biogenesis, dynamics, and turnover) and metabolic functionality. However, much less is known about the mitochondrial role in the pathways that allow a small number of individuals among humans or animals to reach extended longevity [5,6]. Among the few studies comparing old and very old individuals [7,8,9,10], in a recent paper we analyzed mitochondrial markers related to functionality and biogenesis in liver from 28- and 32-month-old rats, highlighting not only relevant differences, but also similarities between the two age groups [11]. One original conclusion driven by the study was that the age-related progressive decline in mitochondrial functionality and biogenesis appeared to proceed at different paces in animals aged 28 months and in those aged 32 months because of the absence of significant differences in several markers between the two groups in spite of the different length of life reached. In particular, we found no decrease in mitochondrial DNA (mtDNA) content or in the amount of the mtDNA histone-like protein mitochondrial transcription factor A (TFAM) in 32-month-old rats compared with those of the 28-month-old animals [11]. Differently from these findings, mtDNA content and TFAM amount were often reported to decrease in various tissues from aged rats (27–28 months old) [12,13,14] and other mammals [15,16] in comparison with younger counterparts. Furthermore, in the same previous study, 32-month-old rats were characterized by close values of fusion index, calculated as Mitofusin 2/Dynamin Related Protein 1 (MFN2/ DRP1) protein ratio, which positively correlated to the respective mtDNA contents [11]. Thus, the suggested mitochondrial contribution to extended longevity implied that a finely-tuned balance of mitochondrial dynamics, together with a “stationary” regulation of mtDNA content and related functional proteins, might have led to the establishment of a permanent “adult-like performance” status in the extremely aged 32-month-old rats. In consideration of such a model, the molecular analysis of some factors controlling mtDNA copy number became very relevant to us. The interaction between TFAM and the mitochondrial genome has been suggested to be involved in the regulation of mtDNA maintenance [17,18,19]. In previous studies, we reported an age-related increase in TFAM binding at regions encompassing the mtDNA replication origins in the liver and soleus muscle of 28-month-old rats [12,20]. More recently, we demonstrated that such region-specific, age-related increase in TFAM binding occurred at mtDNA damage hot spots and was associated with mtDNA loss in the rat heart [14]. To analyze the mitochondrial involvement in rat extended longevity in more depth, we determined various parameters in liver samples from aged (28-month-old) and extremely aged (32-month-old) rats of the naturally long-living Fischer 344 x Brown Norway hybrid strain. We analyzed the 32-month-old rats, considering them as an animal model of human centenarians, which means subjects naturally better adjusted to aging, in which to highlight parameters facilitating extended longevity. The results from the 32-month-old rats have been compared with those from the 28-month-old animals. The age of 28 months has been chosen as one of the last points included in the common pace aging, since after this age survival dramatically decreases according to the mortality curve for this strain [21]. In particular, we compared between the two age groups of rats, selected markers of mitochondrial functionality (activity of citrate synthase) and of mtDNA maintenance, namely amount of TFAM, the content of mtDNA, and of the so-called 4.8 Kb “common deletion” [22,23], together with TFAM binding to mtDNA and the incidence of oxidized purines in specific mtDNA regions. The analyzed regions encompassed the origins of mtDNA replication (D-loop and Ori-L) and the 16-bp long direct repeat 1 (DR1) of the “common deletion” that was chosen as a control region without specific involvement in the replication process. The comparison between aged and extremely aged rats unveiled some unexpected differences for TFAM binding to mtDNA and incidence of oxidized purines that might be relevant for “successful” extended longevity.

## 2. Results

### 2.1. Citrate Synthase Activity, Amount of TFAM, and Contents of mtDNA and “Common Deletion”

**Figure 1:**
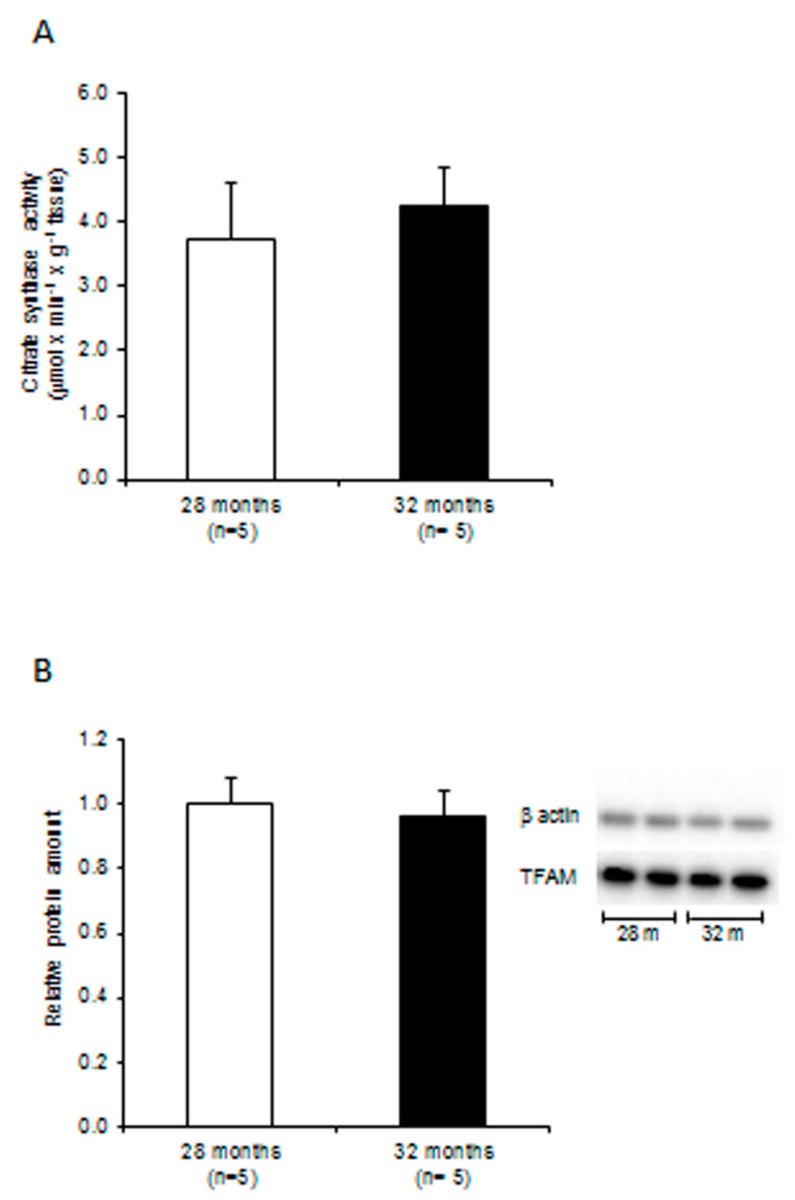
Citrate synthase activity and mitochondrial transcription factor A (TFAM) relative amount in liver from 28- and 32-month-old rats. (**A**) Citrate synthase activity. Bars represent the mean value and SEM of experiments performed in quadruplicate. (**B**) Relative amounts of TFAM. Bars represent the mean and standard error of the mean (SEM) of the relative TFAM/β-actin value, obtained by densitometry analysis of the results from triplicated western blot experiments, from each 28- and 32-month-old rat. The comparison was made with respect to the value of the 28-month-old rats, fixed as 1. Right panel: representative western blot carried out in two rats from each assayed group. The bands, from top to bottom, show the signals from β-actin and TFAM; *n* = number of analyzed animals.

The determination of citrate synthase activity, a marker of mitochondrial mass, in 28- and 32-month-old rats revealed a non-significant 13% increase in the older animals (Figure 1A). The amount of TFAM protein was measured by western blot experiments in both age groups, showing a non-significant 4% reduction (Figure 1B).

**Figure 2:**
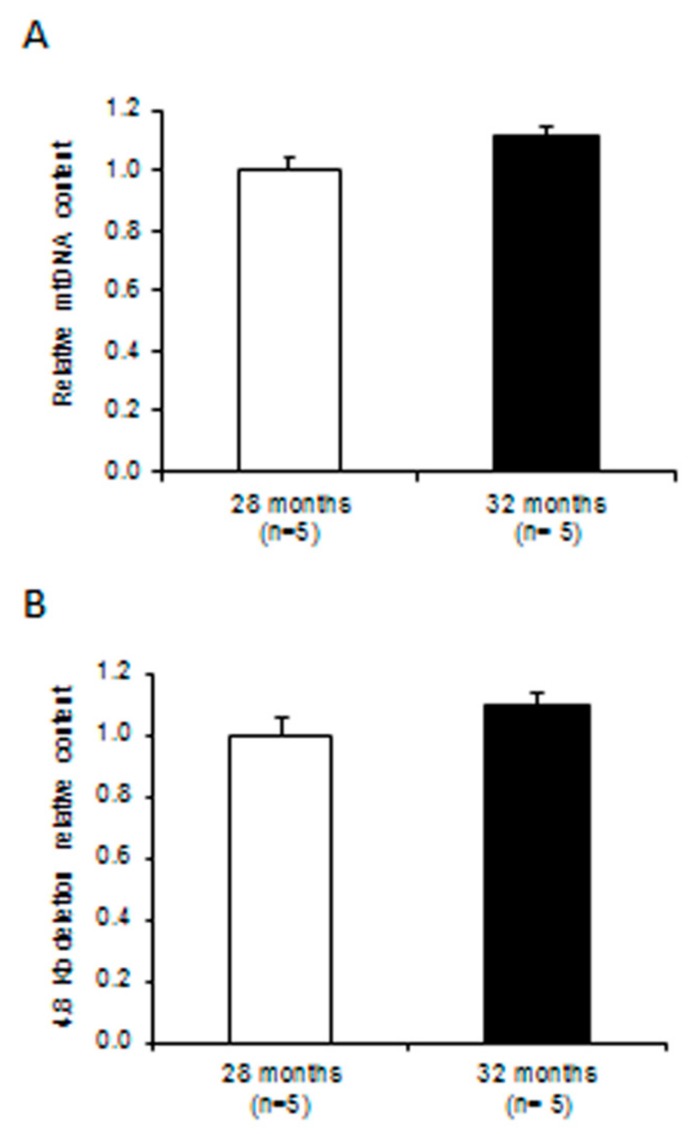
Relative contents of mitochondrial DNA (mtDNA) and of the 4.8 Kb mtDNA deletion in liver from 28- and 32-month-old rats. (**A**) Relative mtDNA content and (**B**) relative 4.8 Kb deletion content determined by quantitative PCR (qPCR). (A,B) Bars represent the mean value and SEM of two independent experiments conducted in triplicate. The comparisons were made with respect to the value of the 28-month-old rats, fixed as 1; *n* = number of analyzed animals.

The non-significant 11% higher mtDNA relative content in 32-month-old rats, as determined by quantitative polymerase chain reaction (qPCR), confirmed the absence of any significant difference in mitochondrial mass between age groups (Figure 2A).

To compare the two rat groups at mtDNA damage level the relative content of the 4.8 Kb “common deletion” was determined by qPCR, showing a non-significant 10% increase in 32-month-old rats (Figure 2B).

### 2.2. TFAM Binding to mtDNA

**Figure 3:**
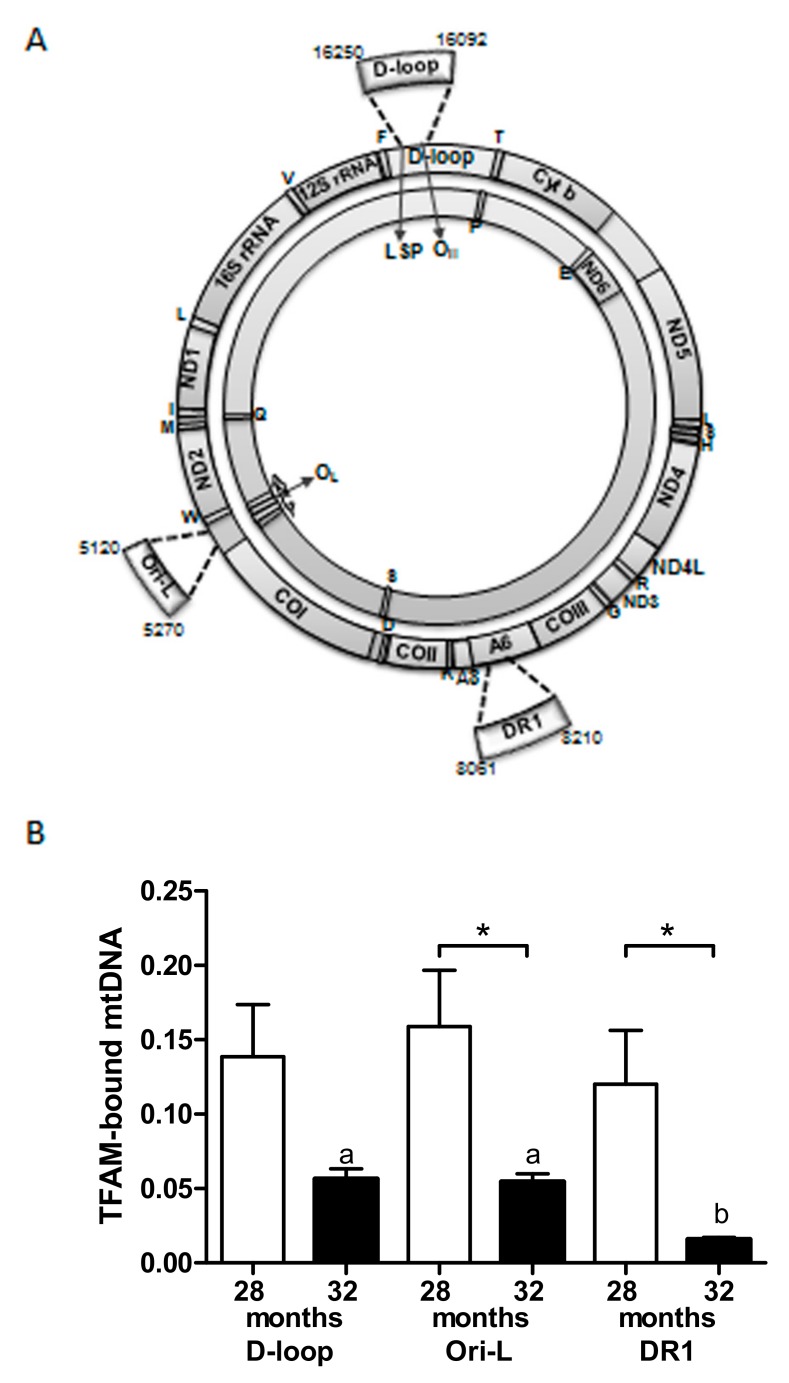
Localization and relative amount of TFAM-bound mtDNA at three mtDNA regions in liver from 28- and 32-month-old rats. (**A**) The amplified products enclose part of D-loop, Ori-L, and DR1-harboring regions, as depicted in the rat mtDNA map. (**B**) Bars represent the mean and SEM of the relative amounts of TFAM-bound mtDNA of two independent experiments performed in triplicate. Number of analyzed animals: 5 in the 28-month-old group, 4 in the 32-month-old group; * *p* < 0.05, Mann Whitney test. Bars not showing the same superscript differ significantly; *p* < 0.01, Dunn’s Multiple comparison test after Kruskal-Wallis test.

The observation of unchanged mtDNA content between aged and extremely aged rats raised questions about the TFAM binding at specific mtDNA regions in the two groups. Therefore, mtDNA immunoprecipitation (mIP) was carried out to screen three regions (Figure 3A)—the two encompassing the mtDNA origins of replication, namely D-loop and Ori-L, and the region including the 16-bp long Direct Repeat 1 (DR1) flanking the 4.8 Kb long “common deletion” of rat mtDNA. The relative amount of TFAM-bound mtDNA, determined by qPCR (Figure 3B), was significantly smaller at Ori-L and DR1 regions (*p* = 0.0434 and *p* = 0.0117, respectively), and approached significance at the D-loop (*p* = 0.0545) in 32-month-old rats in comparison with younger counterparts.

Another interesting result was obtained by comparing the amount of TFAM-bound mtDNA at the three assayed regions in each age group. No differences were observed in 28-month-old rats, whereas older animals showed greater TFAM-bound mtDNA amounts at D-loop and Ori-L regions compared to DR1 values (*p* < 0.01) (Figure 3B).

### 2.3. MtDNA Damage

**Figure 4:**
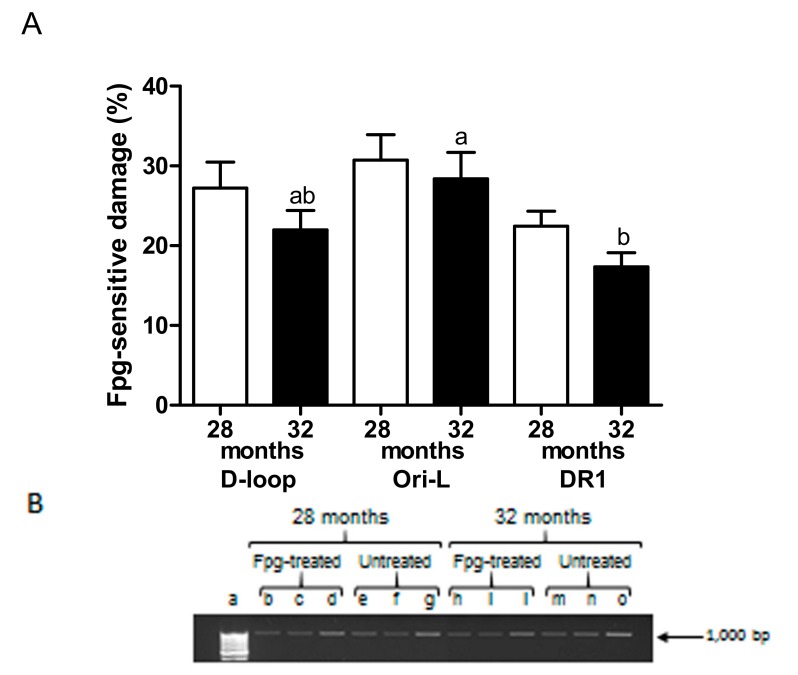
Oxidized purines-specific mtDNA damage at the D-loop, Ori-L, and DR1 regions in liver from 28- and 32-month-old rats. (**A**) Bars represent the mean and SEM of the values obtained for each of the five animals analyzed in both groups. Bars not showing the same superscript differ significantly; *p* < 0.05, Dunn’s Multiple comparison test after Kruskal-Wallis test. (**B**) Representative gel of formamidopyrimidine DNA glycosylase (Fpg)-treated and untreated total DNA from a 28-month-old rat and a 32-month-old animal; 2.5 and 5 ng total DNA were amplified using the D-loop long primer set (Table 1). An aliquot of each PCR amplification was loaded onto agarose ethidium bromide-stained gel and analyzed for band intensities (a: molecular weight marker (GeneRuler 100 bp DNA Ladder, Thermo Fisher Scientific); b, c, e, f, h, i, m, and n: 2.5 ng total DNA as PCR template; d, g, l, and o: 5 ng total DNA as PCR template).

In order to unveil a possible molecular cause for reduced TFAM binding at the analyzed regions in older animals, the same three regions were screened for the incidence of oxidized purines, mainly 8-oxo-deoxyguanosine (8-oxodG), using the oxidized purines-sensitive enzyme formamidopyrimidine DNA glycosylase (Fpg) [4,14]. A trend towards reduced incidence of oxidized purines at all assayed regions was observed in 32-month-old rats compared to the younger counterparts (Figure 4A). Furthermore, there was no significant difference as for incidence of oxidized purines when the three regions were compared in the 28-month-old rats, while in the 32-month-old animals such a difference was present, and the Dunn’s multiple comparison test showed a significant difference between Ori-L and DR1 regions (*p* < 0.05; Figure 4A).

**Figure 5:**
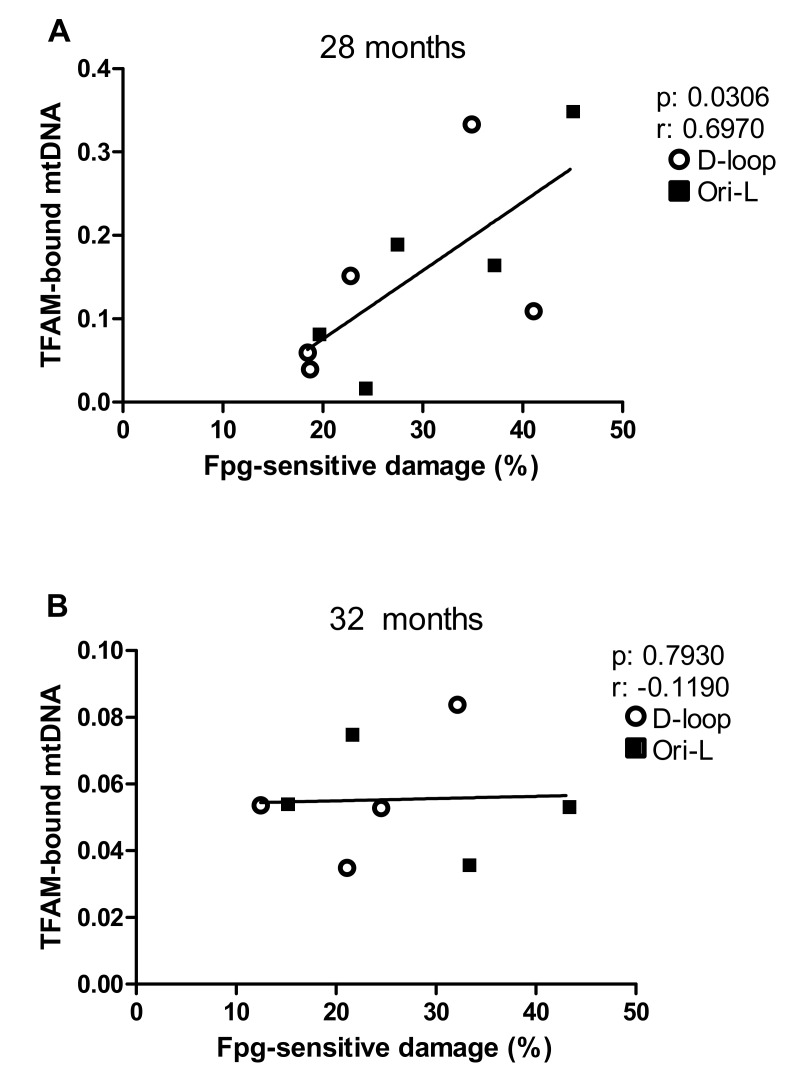
Correlation analysis between Fpg-sensitive damage and TFAM-bound mtDNA amounts at the D-loop and Ori-L regions by age groups. Data in the graph are mean values, obtained at the D-loop and Ori-L regions, respectively, from each analyzed rat. Number of analyzed animals: 5 in the 28-month-old group, 4 in the 32-month-old group; p, r = Spearman correlation test.

Therefore, we explored the relationship between the incidence of Fpg-sensitive damages and TFAM-bound mtDNA amount at the two regions encompassing the replication origins in each age group. The Spearman correlation analysis revealed a significant positive correlation between the incidence of oxidized purines and the amount of TFAM-bound mtDNA in 28-month-old rats (*p* = 0.0306; *r* = 0.6970, Figure 5A), but not in the 32-month-old rats (Figure 5B).

## 3. Discussion

In order to dissect the multiple determinants of aging, it is very effective to carry out not only the analysis of the changes characterizing the progression from young to old age, but also the examination of differences between aged individuals and “successfully” extremely aged subjects. The few studies comparing aged (28–30–-month-old) and senescent (35–37–-month-old) rats for different cellular and mitochondrial markers in skeletal muscle usually assessed senescent rats as the final stage of a continuous linear aging process that should have led to the most severe dysfunctional changes [7,8,9,10]. However, there is no guarantee about the linearity of the aging process [24], and therefore, according to our previous results [11], we considered those rats reaching extended longevity as the animal counterpart of human centenarians, which means subjects who did grow old as for general and cellular evaluation, but that were “successfully” aged, being still able to cope with several situations in a way typical of younger ones [25,26,27]. We evaluated the age of 32 months as sufficiently adequate to highlight those mitochondrial parameters facilitating extended longevity, without the need to analyze older animals.

As clearly stated by Gondo et al. [24], the issue of the control group in studies about centenarians is crucial. Indeed, such a group should allow differentiation between age-related changes and factors influencing longevity. If the control group were made up of young subjects, only the age-related changes would be evident in the comparison with the extremely aged counterparts, whereas a control group constituted by aged subjects would better highlight differences and similarities involved in maintaining healthy longevity. We studied liver from aged (28 months) rats, which were identified as the end of the common pace aging, and extremely aged (32 months old) rodents, which were evaluated as somehow able to counteract this process, slowing its pace, and thus, overall, being better adjusted to aging. In particular, we analyzed at the molecular level some features of mtDNA maintenance involved in mitochondrial biogenesis, which is affected by aging [12,20].

Indeed, the aged rat liver shows a marked decrease in mitochondrial functionality [28] and alterations of mitochondrial biogenesis [20]. Here, mitochondrial functionality was assayed through the determination of citrate synthase activity, which did not significantly change between the two age groups. This evidence suggests that the decline of citrate synthase activity, usually reported from young to old age [29,30], might have proceeded at a slower pace in the successfully extremely aged, in which citrate synthase activity was not different from 28-month-old rodents. Mitochondrial biogenesis was analyzed through the determination of TFAM amount and mtDNA content, revealing again no significant differences between 28- and 32-month-old rats. Thus, also the age-related progressive loss of TFAM amount [20] and mtDNA content [12,13,14,15,16] seemed to proceed at a slower pace in the 32-month-old animals. Such data, together with the unchanged activity of citrate synthase, suggest that in those rats, which reached 32 months, the age-related mitochondrial dysfunction occurred at a slower pace, inducing less severe damages than expected for functionality and mtDNA maintenance. The evaluation of the mtDNA 4.8 Kb deletion content showed a non-significant change between the two age groups, which is remarkably different compared with the 40–100-fold increase described in passing from young to old rats [22,31]. This finding further supports the idea that a “stationary” regulation of mtDNA content and quality, as well as of related functional proteins, is peculiarly established in the successfully extremely aged rats.

Contrary to the above-reported similarities, the determination of the amount of TFAM-bound mtDNA revealed the clear reduction of TFAM binding at D-loop, Ori-L, and DR1 regions in 32-month-old rats in comparison with 28-month-old ones. The decreased TFAM binding cannot be explained by reduced TFAM protein expression, because there was no significant difference between age groups. In the framework of the overall reduction of TFAM binding in 32 months rats, significantly higher values were found at the D-loop and Ori-L regions than at the respective DR1 counterpart. Such differences suggest that the TFAM binding at the regions encompassing the replication origins allowed an adequate mtDNA maintenance in successfully extremely aged rats. In contrast, when a sort of “threshold” was exceeded, the binding of TFAM might have become excessive and have prevented, rather than facilitated, mtDNA replication [20]. It is worth mentioning that previous “in vitro” studies demonstrated that different TFAM concentrations and sequential binding to mtDNA induced opposing effects on initiation of transcription at the heavy strand promoter 2 (HSP2) region [32,33,34]. The present data suggest that TFAM binding to mtDNA might be modulated in different ways in aging and in extended longevity.

An age-related accumulation of oxidized purines, especially 8-oxodG, has been described in various rat tissues [35,36,37]. Therefore, we screened the above-mentioned mtDNA regions using the oxidized purines-sensitive enzyme Fpg. The trend towards a reduced incidence of oxidized purines at all assayed regions in the 32-month-old group with respect to the 28-month-old rats was analogous to the TFAM binding results. The more relevant 8-oxodG presence in 28-month-old rats cannot be attributed to a decrease in activity [38] or in expression [11] of mitochondrial 8-oxoguanine DNA glycosylase (OGG1). Rather, it suggests a more severe oxidative damage in this age group. Interestingly, in the overall reduced presence of oxidized purines in the 32 months old rats, higher values of Fpg-sensitive damage were found at D-loop and Ori-L in comparison with the respective value at DR1. This finding allowed us to identify the regions encompassing the replication origins as hot spots for oxidative damage, similarly to what we found in aged rat heart [14].

Recent studies demonstrated that differences in TFAM binding were linked to changes in mtDNA damage [14,39], and therefore, we searched this kind of relationship at the regions encompassing the replication origins in both age groups. Interestingly, the present results show a significant positive correlation between Fpg-sensitive damage and TFAM-bound mtDNA at the two assayed regions in 28-month-old rats. These findings suggest that in this age-group, extensive oxidative damage might have led to increased, perhaps excessive, TFAM binding, such as to hamper efficient repair/replication processes, possibly contributing to age-related mtDNA loss [14,40]. In contrast, such a correlation was not found in 32-month-old rats. This may indicate that in extremely old rats, a lower burden of oxidative damage did not induce an increase in TFAM binding, therefore allowing efficient repair/replication processes and maintenance of mtDNA content.

## 4. Materials and Methods

### 4.1. Animals

The Institutional Animal Care and Use Committee at the University of Florida (Gainesville, FL, USA) approved the study protocol. All procedures were conducted according to the National Institutes of Health guidelines for the care and use of laboratory animals. Liver samples were obtained from Fischer 344 × Brown Norway (F344BNF1) male rats purchased from the National Institute of Aging colony (Indianapolis, IN, USA) and housed at the Department of Aging and Geriatric Research, Division of Biology of Aging, College of Medicine, University of Florida (Gainesville, FL, USA), in the institutional animal housing facility. According to the study design, animals were subdivided into two groups: rats belonging to the first group were sacrificed at the age of 28 months (*n* = 5), and rats belonging to second group were sacrificed at the age of 32 months (*n* = 5). The initial size of the age-groups was larger than the final one to compensate for intervening natural mortality. The rats were housed individually in a temperature- (20 +/−2 °C) and light-controlled environment (12-h light/dark cycle) with regular rat chow and water available *ad libitum*. Rats were anesthetized before being sacrificed and the liver was immediately removed, snap-frozen in isopentane cooled by liquid nitrogen, and stored in liquid nitrogen until further analysis.

### 4.2. Determination of Citrate Synthase Activity

Total proteins were purified from ∼50 mg of liver samples by homogenization in a buffer containing 100 mM mannitol, 1 mM ATP, 0.2% bovine serum albumin (BSA), 100 mM KCl, 3 mM MgCl_2_, 5 mM Tris-buffer, 1 mM EDTA, pH 7.4. Protein concentration was determined by the Bradford method [41] according to the supplier’s instructions (Bio-Rad Laboratories Inc., Hercules, CA, USA).

Citrate synthase activity (μmol × min^−1^ × g tissue^–1^) was measured. Briefly, 80 μg of total proteins were incubated in 1 mL assay buffer containing 0.31 mM acetyl-CoA, 100 mM Tris buffer (pH 8.1), 0.25% Triton X-100, 0.1 mM 550-dithio-bis-2-nitrobenzoic acid, and 0.5 mM oxaloacetate at 30 °C. Citrate synthase activity was determined spectrophotometrically by measuring the rate of production of thionitrobenzoic acid (TNB) at 412 nm [42].

### 4.3. Western Blots

Total protein extracts measuring 10 μg from each sample in 5× Laemmli sample buffer were denatured and loaded into 4–12% pre-cast polyacrylamide gels (Bio-Rad, Milan, Italy). Custom made [20] rabbit anti-TFAM (1:30,000) and anti-β-actin (A2066, Sigma Aldrich, Milan, Italy) (1:20,000) were used as primary antibodies. Membranes were further incubated with a horseradish peroxidase-conjugated rabbit secondary antibody. The proteins were detected by chemiluminescence (Clarity Western ECL substrate, Bio-Rad, Milan, Italy). Signals were analyzed by laser densitometry with the Chemi Doc System and Image Lab software (Bio-Rad Laboratories Inc., Hercules, CA, USA). The densitometric value (OD units) of each band was then normalized against β-actin expression.

### 4.4. Determination of mtDNA and mtDNA 4.8 Kb Deletion Content

Quantitative real time polymerase chain reaction (qPCR) was used to determine relative contents of mtDNA and mtDNA 4,834 bp (4.8 Kb) deletion. Reactions were performed via SYBR Green chemistry using 3 ng total DNA as template. MtDNA content (mtDNA primer set) relative to nuclear DNA (β-actin primer set) was measured as previously reported [11]. The relative content of the 4.8 Kb deletion (4.8 Del primer set) normalized to mtDNA content was determined according to the formula 2^ΔCTx^ − 2^ΔCTb^ (Δ^CTx^ is the difference between values of 4.8 Del CT values and mtDNA CT, and Δ^CTb^ is the difference between the mtDNA CT values and β-actin CT). Primer sequences are reported in Table 1.

### 4.5. Mitochondrial DNA Immunoprecipitation (mIP) and Quantitative PCR of mIP DNA

The binding of TFAM to specific regions of mtDNA was analyzed using mtDNA immunoprecipitation (mIP) following the procedure described elsewhere [43]. Due to the extremely small size of the tissue samples the mIP analysis was not performed in one out of the five 32-month-old animals.

The determination of the relative content of TFAM-immunoprecipitated mtDNA was carried out by qPCR. To analyze the D-loop, Ori-L, and DR1 mtDNA regions bound by TFAM, RT D-loop, RT Ori-L, and RT DR1 primer sets (Table 1) and 2.5 μL of the input, or the immunoprecipitated with anti-TFAM or without antibody DNA aliquots, respectively, were used. The qPCR reactions were performed via SYBR Green chemistry. The calculation of the relative content of TFAM-bound mtDNA was performed according to the formula 2^ΔCTx^ − 2^ΔCTb^ [44] for each analyzed region.

### 4.6. Analysis of Modified Purines

Formamidopyrimidine DNA glycosylase (Fpg) (New England Biolabs, Beverly, MA, USA) digestion of total DNA was used to detect oxidized purines, according to Pastukh et al. [39]. PCR amplification of the D-loop, Ori-L, and DR1 regions of mtDNA was conducted using the respective long primer sets (Table 1) on 2.5 and 5 ng of Fpg-treated and untreated total DNA. The cycling conditions were: pre-incubation of 10 min at 95 °C, followed by 18 cycles of 15 s 95 °C, 15 s 58 °C, and 1 min 72 °C. An aliquot of each PCR amplification was loaded on 1.3% agarose gel. Ethidium bromide-stained bands were visualized and band intensities were analyzed by Image Lab Software (BioRad Laboratories Inc., Hercules, CA, USA). To improve graphical visualization, the ratio between Fpg-treated and untreated band intensities was expressed as percentage of the complement to 100.

### 4.7. Statistics

Data are expressed as mean and standard error of the mean (SEM). The Mann Whitney test or the Kruskal-Wallis with Dunn’s multiple comparison test was used when appropriate. Correlations between variables were determined by Spearman statistics. For all tests, statistical significance was set at 5% level. Analyses were run using a specific statistical package (Stata Corp. 2005. Stata Statistical Software: Release. College Station, TX, USA).

## 5. Conclusions

The results here reported support the existence of a fine-tuned regulation of TFAM binding in successfully extremely aged rats, such that repair, degradation, and synthesis of mtDNA remain balanced. Such balance, leading to improved mtDNA maintenance in extended aging, might involve the presence of stress signals, sent through mtDNA occupancy and damage at crucial functional regions of the molecule, which impinge on nuclear regulation of expression and whole cell responses. Further studies are needed to corroborate the hypothesis of a peculiar pace in successfully extended aging, different from that in aging. Having assessed this, approaches to establish and maintain the features of the successful “extended longevity” might be pursued.

## Figures and Tables

**Table 1:** Oligonucleotide primer sequences.

Primer Set	Forward Primer	Reverse Primer	(nps)	(nps)
mtDNA	5′GGTTCTTACTTCAGGGCCATCA3′	5′TGATTAGACCCGTTACCATCGA3′	15,785–15,806	15,868–15,847
β-actin	5′CCCAGCCATGTACGTAGCCA3′	5′CGTCTCCGGAGTCCATCAC3′	2181–2200	2266–2248
4.8 Del	5′AAGGACGAACCTGAGCCCTAATA3′	5′CGAAGTAGATGATGCGTATACTGTA3′	8109–8131	13,020–12,996
RT D-loop	5′CACCCCCTACACCTGAAACTT3′	5′TTTGTGTCGGGAAATTTTACCAAT3′	16,092–16,112	16,250–16,227
RT Ori-L	5′CAGCTAAATACCCTACTTACTGG3′	5′GCCCCCTTTTTACCAAAAAGCC3′	5120–5142	5270–5249
RT DR1	5′GACTAATCAAACTTATCATCAAACAA3′	5′ GCTGAGTGGTAGGGGTAAATGT3′	8061–8086	8210–8189
D-loop long	5′TCTGGTCTTGTAAACCAAAAATGA3′	5′TGGAATTTTCTGAGGGTAGGC3′	15,302–15,325	16,302–16,282
Ori-L long	5′AACCAGACCCAAACACGAAA3′	5′CTATTCCTGCTCAGGCTCCA3′	4414–4433	5407–5388
DR1 long	5′CCGCCTAAACCAAGCTACAG3′	5′AAGGCTACGGCAAATTCAAG’	7536–7555	8541–8522

Numbering is according to GenBank™ accession number AY172581 (Rattus norvegicus, complete mitochondrial genome), except for the β-actin primer set, which is according to GenBank™ accession number V01217.1 (Rattus norvegicus, β-actin gene); nps: nucleotide positions.

## Abbreviations

Fpg	Formamidopyrimidine DNA glycosylase

## References

[1] López-Otín C., Blasco M.A., Partridge L., Serrano M., Kroemer G. (2013). The hallmarks of aging. Cell.

[2] Held N.M., Houtkooper R.H. (2015). Mitochondrial quality control pathways as determinants of metabolic health. Bioessays.

[3] López-Lluch G., Santos-Ocaña C., Sánchez-Alcázar J.A., Fernández-Ayala D.J., Asencio-Salcedo C., Rodríguez-Aguilera J.C., Navas P. (2015). Mitochondrial responsibility in ageing process: Innocent, suspect or guilty. Biogerontology.

[4] Picca A., Sirago G., Pesce V., Lezza A.M.S., Calvani R., Bossola M., Villani E.R., Landi F., Leeuwenburgh C., Bernabei R. (2018). Administration of Enalapril Started Late in Life Attenuates Hypertrophy and Oxidative Stress Burden, Increases Mitochondrial Mass, and Modulates Mitochondrial Quality Control Signaling in the Rat Heart. Biomolecules.

[5] De Benedictis G., Rose G., Carrieri G., De Luca M., Falcone E., Passarino G., Bonafe M., Monti D., Baggio G., Bertolini S. (1999). Mitochondrial DNA inherited variants are associated with successful aging and longevity in humans. FASEB J..

[6] Kolovou G.D., Kolovou V., Mavrogeni S. (2014). We are ageing. Biomed. Res. Int..

[7] Marzetti E., Eva S., Anne H., Chung H., Giovannini S., Leeuwenburgh C. (2008). Age-related activation of mitochondrial caspase-independent apoptotic signaling in rat gastrocnemius muscle. Mech. Ageing Dev..

[8] Baker D.J., Hepple R.T. (2006). Elevated caspase and AIF gene expression correlate with progression of sarcopenia during aging in male F344BN rats. Exp. Gerontol..

[9] Hepple R.T., Hagen J.L., Krause D.J., Baker D.J. (2004). Skeletal muscle aging in F344BN F1-hybrid rats: II. Improved contractile economy in senescence helps compensate for reduced ATP-generating capacity. J. Gerontol. A Biol. Sci. Med. Sci..

[10] Hagen J.L., Krause D.J., Baker D.J., Fu M.H., Tarnopolsky M.A., Hepple R.T. (2004). Skeletal muscle aging in F344BN F1-hybrid rats: I. Mitochondrial dysfunction contributes to the age-associated reduction in VO2max. J. Gerontol. A Biol. Sci. Med. Sci..

[11] Picca A., Pesce V., Sirago G., Fracasso F., Leeuwenburgh C., Lezza A.M.S. (2016). “What makes some rats live so long?” The mitochondrial contribution to longevity through balance of mitochondrial dynamics and mtDNA content. Exp. Gerontol..

[12] Picca A., Pesce V., Fracasso F., Joseph A.M., Leeuwenburgh C., Lezza A.M. (2014). A comparison among the tissue-specific effects of aging and calorie restriction on TFAM amount and TFAM-binding activity to mtDNA in rat. Biochim. Biophys. Acta.

[13] Nicassio L., Fracasso F., Sirago G., Musicco C., Picca A., Marzetti E., Calvani R., Cantatore P., Gadaleta M.N., Pesce V. (2017). Dietary supplementation with acetyl-l-carnitine counteracts age-related alterations of mitochondrial biogenesis, dynamics and antioxidant defenses in brain of old rats. Exp. Gerontol..

[14] Chimienti G., Picca A., Sirago G., Fracasso F., Calvani R., Bernabei R., Russo F., Carter C.S., Leeuwenburgh C., Pesce V. (2018). Increased TFAM binding to mtDNA damage hot spots is associated with mtDNA loss in aged rat heart. Free Radic. Biol. Med..

[15] Mengel-From J., Thinggaard M., Dalgard C., Kyvik K.O., Christensen K., Christiansen L. (2014). Mitochondrial DNA copy number in peripheral blood cells declines with age and is associated with general health among elderly. Hum. Genet..

[16] Li C., White S.H., Warren L.K., Wohlgemuth S.E. (2018). Skeletal muscle from aged American Quarter Horses shows impairments in mitochondrial biogenesis and expression of autophagy markers. Exp. Gerontol..

[17] Clay Montier L.L., Deng J.J., Bai Y. (2009). Number matters: Control of mammalian mitochondrial DNA copy number. J. Genet. Genomics.

[18] Campbell C.T., Kolesar J.E., Kaufman B.A. (2012). Mitochondrial transcription factor A regulates mitochondrial transcription initiation, DNA packaging, and genome copy number. Biochim. Biophys. Acta.

[19] Picca A., Lezza A.M. (2015). Regulation of mitochondrial biogenesis through TFAM-mitochondrial DNA interactions: Useful insights from aging and calorie restriction studies. Mitochondrion.

[20] Picca A., Pesce V., Fracasso F., Joseph A.M., Leeuwenburgh C., Lezza A.M.S. (2013). Aging and calorie restriction oppositely affect mitochondrial biogenesis through TFAM binding at both origins of mitochondrial DNA replication in rat liver. PLoS ONE.

[21] Turturro A., Witt W.W., Lewis S., Hass B.S., Lipman R.D., Hart R.W. (1999). Growth curves and survival characteristics of the animals used in the Biomarkers of Aging Program. J. Gerontol. A Biol. Sci. Med. Sci..

[22] Gadaleta M.N., Rainaldi G., Lezza A.M., Milella F., Fracasso F., Cantatore P. (1992). Mitochondrial DNA copy number and mitochondrial DNA deletion in adult and senescent rats. Mutat. Res..

[23] Yowe D.L., Ames B.N. (1998). Quantitation of age-related mitochondrial DNA deletions in rat tissues shows that their pattern of accumulation differs from that of humans. Gene.

[24] Gondo Y., Masui Y., Kamide K., Ikebe K., Arai Y., Ishizaki T., Pachana N.A. (2016). SONIC Study. Encyclopedia of Geropsychology.

[25] Puca A.A., Spinelli C., Accardi G., Villa F., Caruso C. (2018). Centenarians as a model to discover genetic and epigenetic signatures of healthy ageing. Mech. Ageing Dev..

[26] Khan S.S., Singer B.D., Vaughan D.E. (2017). Molecular and physiological manifestations and measurement of aging in humans. Aging Cell..

[27] Shadyab A.H., LaCroix A.Z. (2015). Genetic factors associated with longevity: A review of recent findings. Ageing Res. Rev..

[28] Navarro A., Boveris A. (2007). The mitochondrial energy transduction system and the aging process. Am. J. Physiol. Cell Physiol..

[29] Pesce V., Nicassio L., Fracasso F., Musicco C., Cantatore P., Gadaleta M.N. (2012). Acetyl-L-carnitine activates the peroxisome proliferator-activated receptor-γ coactivators PGC-1α/PGC-1β-dependent signaling cascade of mitochondrial biogenesis and decreases the oxidized peroxiredoxins content in old rat liver. Rejuvenation Res..

[30] Hebert S.L., Marquet-de Rougé P., Lanza I.R., McCrady-Spitzer S.K., Levine J.A., Middha S., Carter R.E., Klaus K.A., Therneau T.M., Highsmith E.W. (2015). Mitochondrial Aging and Physical Decline: Insights From Three Generations of Women. J. Gerontol. A Biol. Sci. Med. Sci..

[31] Edris W., Burgett B., Stine O.C., Filburn C.R. (1994). Detection and quantitation by competitive PCR of an age-associated increase in a 4.8-kb deletion in rat mitochondrial DNA. Mutat. Res..

[32] Lodeiro M.F., Uchida A., Bestwick M., Moustafa I.M., Arnold J.J., Shadel G.S., Cameron C.E. (2012). Transcription from the second heavy-strand promoter of human mtDNA is repressed by transcription factor A in vitro. Proc. Natl. Acad. Sci. USA.

[33] Zollo O., Tiranti V., Sondheimer N. (2012). Transcriptional requirements of the distal heavy-strand promoter of mtDNA. Proc. Natl. Acad. Sci. USA.

[34] Shutt T.E., Bestwick M., Shadel G.S. (2011). The core human mitochondrial transcription initiation complex. It only takes two to tango. Transcription.

[35] Hudson E.K., Hogue B.A., Souza-Pinto N.C., Croteau D.L., Anson R.M., Bohr V.A., Hansford R.G. (1998). Age-associated change in mitochondrial DNA damage. Free Radic. Res..

[36] López-Torres M., Gredilla R., Sanz A., Barja G. (2002). Influence of aging and long-term caloric restriction on oxygen radical generation and oxidative DNA damage in rat liver mitochondria. Free Radic. Biol. Med..

[37] Nakamoto H., Kaneko T., Tahara S., Hayashi E., Naito H., Radak Z., Goto S. (2007). Regular exercise reduces 8-oxodG in the nuclear and mitochondrial DNA and modulates the DNA repair activity in the liver of old rats. Exp. Gerontol..

[38] Souza-Pinto N.C., Croteau D.L., Hudson E.K., Hansford R.G., Bohr V.A. (1999). Age-associated increase in 8-oxo-deoxyguanosine glycosylase/AP lyase activity in rat mitochondria. Nucleic Acids Res..

[39] Pastukh V.M., Gorodnya O.M., Gillespie M.N., Ruchko M.V. (2016). Regulation of mitochondrial genome replication by hypoxia: The role of DNA oxidation in D-loop region. Free Radic. Biol. Med..

[40] Caston R.A., Demple B. (2017). Risky repair: DNA-protein crosslinks formed by mitochondrial base excision DNA repair enzymes acting on free radical lesions. Free Radic. Biol. Med..

[41] Bradford M.M. (1976). A rapid and sensitive method for the quantitation of microgram quantities of protein utilizing the principle of protein-dye binding. Anal. Biochem..

[42] Srere P.A. (1969). Citrate synthase: [EC 4.1.3.7. Citrate oxaloacetate-lyase (CoA-acetylating)]. Methods Enzymol..

[43] Picca A., Fracasso F., Pesce V., Cantatore P., Joseph A.M., Leeuwenburgh C., Gadaleta M.N., Lezza A.M. (2013). Age- and calorie restriction-related changes in rat brain mitochondrial DNA and TFAM binding. Age.

[44] Vercauteren K., Pasko R.A., Gleyzer N., Marino V.M., Scarpulla R.C. (2006). PGC-1-related coactivator: Immediate early expression and characterization of a CREB/NRF-1 binding domain associated with cytochrome c promoter occupancy and respiratory growth. Mol. Cell. Biol..

